# Comparative Usability Analysis and Parental Preferences of Three Web-Based Knowledge Translation Tools: Multimethod Study

**DOI:** 10.2196/14562

**Published:** 2020-03-13

**Authors:** Harrison Anzinger, Sarah Alexandra Elliott, Lisa Hartling

**Affiliations:** 1 Department of Pediatrics University of Alberta Edmonton, AB Canada; 2 Cochrane Child Health Edmonton, AB Canada

**Keywords:** child health, knowledge translation, parents, internet, comparative study, health information,consumer

## Abstract

**Background:**

Connecting parents to research evidence is known to improve health decision making. However, guidance on how to develop effective knowledge translation (KT) tools that synthesize child-health evidence into a form understandable by parents is lacking.

**Objective:**

The aim of this study was to conduct a comparative usability analysis of three Web-based KT tools to identify differences in tool effectiveness, identify which format parents prefer, and better understand what factors affect usability for parents.

**Methods:**

We evaluated a Cochrane plain language summary (PLS), Blogshot, and a Wikipedia page on a specific child-health topic (acute otitis media). A mixed method approach was used involving a knowledge test, written usability questionnaire, and a semistructured interview. Differences in knowledge and usability questionnaire scores for each of the KT tools were analyzed using Kruskal-Wallis tests, considering a critical significance value of *P*=.05. Thematic analysis was used to synthesize and identify common parent preferences among the semistructured interviews. Key elements parents wanted in a KT tool were derived through author consensus using questionnaire data and parent interviews.

**Results:**

In total, 16 parents (9 female) with a mean age of 39.6 (SD 11.9) years completed the study. Parents preferred the Blogshot over the PLS and Wikipedia page (*P*=.002) and found the Blogshot to be the most aesthetic (*P*=.001) and easiest to use (*P*=.001). Knowledge questions and usability survey data also indicated that the Blogshot was the most preferred and effective KT tool at relaying information about the topic. Four key themes were derived from thematic analysis, describing elements parents valued in KT tools. Parents wanted tools that were (1) simple, (2) quick to access and use, and (3) trustworthy, and which (4) informed how to manage the condition. Out of the three KT tools assessed, Blogshots were the most preferred tool by parents and encompassed these four key elements.

**Conclusions:**

It is important that child health evidence be available in formats accessible and understandable by parents to improve decision making, use of health care resources, and health outcomes. Further usability testing of different KT tools should be conducted involving broader populations and other conditions (eg, acute vs chronic) to generate guidelines to improve KT tools for parents.

## Introduction

### Background

The impact of health research is often minimized by ineffective communication and utilization. Knowledge translation (KT) offers a solution to this problem through raising awareness of evidence and facilitating its use. Impactful KT goes beyond dissemination, and involves engagement, participation, and evaluation by knowledge users [1]. Cochrane Child Health seeks to facilitate the uptake of evidence and evidence-informed decisions by key knowledge users (clinicians, patients, parents, and policy makers) and other stakeholders (eg, health system organizations such as Alberta Health Services) through the development and dissemination of a variety of engaging KT tools. Owing to the unique needs of pediatric patients, Cochrane Child Health aims to develop parent- and guardian-directed KT tools to meet their needs. Although we know connecting parents and caregivers to research evidence has the power to improve health decision making and appropriate access of health care services [2], traditional KT tools directed at health care professionals remain too complex for parents to effectively utilize [3]. Although many previous attempts have been made to create more user-friendly KT tools, finding a method of realizing this goal has been a persistent difficulty in the child health field.

Usability is a concept developed in the software and Web design industry that is increasingly being applied to KT tools to systematically address factors limiting a KT tool from meeting the needs of its audience. Usability aims to develop tools that provide relevant information in a satisfying, effective, and efficient way to the target end user [3]. At the center of improving usability is field testing and iterative design, which has been adopted by the KT field to develop more user-centered tools for consumers [4-8]. Through this method, KT tools are developed, evaluated, redesigned, and reevaluated based on feedback from the end user. Comparative usability analysis offers another method of assessing the usability of a product. In comparative usability analysis, several prototypes or competing products are compared with one another to identify strengths and weaknesses between the products. Allowing participants to see multiple designs allows them to provide comparative feedback and identify specific areas they like or dislike about a product. Doing such work early in design provides an opportunity to create an end product using the best features from each tool and to better understand the underlying concepts leading to end users’ preferences for a specific product. Although iterative design has been readily adopted into the KT research, comparative usability analysis remains unused in the broader usability field.

Despite usability importance and integration, many KT tools remain complicated and inaccessible to parents and caregivers, creating a drought of understandable knowledge on many topics. As a result, internet KT tools have emerged as a popular source to fill the public demand for understandable, accessible health care knowledge, with 69% of Canadians reporting using the internet for health-related information [9]. Health information on the internet is used in a multitude of ways, including as a second opinion, to determine when to access care, and to inform lifestyle changes [10]. However, with such a wide range of information available over Web, the quality of health information is often mixed and inaccurate [11,12].

In recent years, Cochrane initiatives have aimed to address this need by developing quality KT tools that are available to consumers over Web. Despite Cochrane systematic reviews being regarded as providing the highest quality evidence to make informed choices about health care treatment; they are often inaccessible and impenetrable to parents and familial caregivers. Cochrane has addressed these usability concerns by developing several consumer-orientated KT tools. There are three such tools: the plain language summaries (PLS), Blogshots, and updated Wikipedia pages through the Cochrane-Wikipedia partnership.

### Objectives

Although PLS, Blogshots, and related Wikipedia pages on child health–related topics are readily available to the public, little is known about their usability to parents and familial caregivers. Specifically, we wanted to know more about parents’ views of these tools to further increase their usability and inform future KT tool creation. Subsequently, we conducted a comparative usability analysis of three Web-based Cochrane KT tools to identify differences in tool effectiveness, identify which format parents prefer, and better understand what factors affect usability for parents.

## Methods

### Overview

A mixed method study comprising a knowledge test, written questionnaire, and semistructured interview was conducted to assess the usability of a child health–related Cochrane PLS, Blogshot (developed by Cochrane Child Health), and Wikipedia page on the topic *acute otitis media* (AOM). Study participants were randomly assigned one KT tool to evaluate.

**Figure 1:**
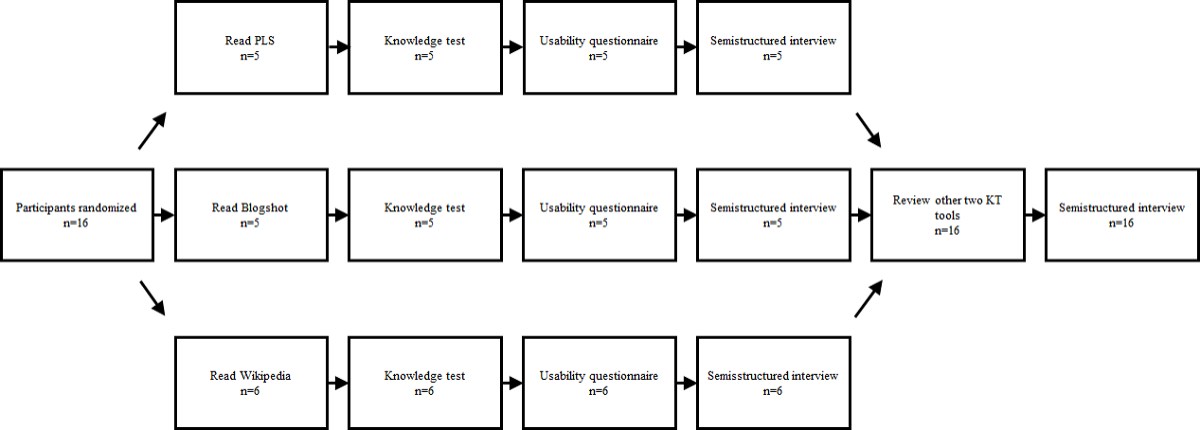
Participant flow diagram. Participants (n=16) were randomized to 1 of 3 groups: Cochrane plain language summary (n=5), Cochrane Blogshot (n=5), or Wikipedia Page (n=6). Participants had unlimited time to read the knowledge translation (KT) tool. Participants were then given a knowledge test without being able to refer back to the KT tool. Participants then completed a written usability questionnaire and semistructured interview focusing on the KT tool randomly assigned. Finally, participants were asked to read the other two KT tools, and a second semistructured interview was completed focusing on comparing the three tools and broader participant preferences for KT tools. All participants completed the study. KT: knowledge translation; PLS: plain language summary.

Interview design is demonstrated in the participant flow diagram (Figure 1). Briefly, participants were given a situational prompt and instructed to read or skim the KT tool as they would if they suspected their child had an ear infection. Participants had as long as they wanted to read the KT tool, and reading time was recorded without the participant’s knowledge as a measure of tool efficiency. As we define usability as a tool that is satisfying, effective, and efficient to the end user, we felt that recording reading time was an important marker of efficiency [3]. The participants were given a structured questionnaire (including a knowledge test) about the KT tool they had just read (Multimedia Appendix 1). Participants then took part in a one-on-one interview. Half way through the interview, participants were shown the two other KT tools for comparison. Participants were then asked to rank the three KT tools in order of aesthetics, ease of use, credibility, and general preference.

**Figure 2:**
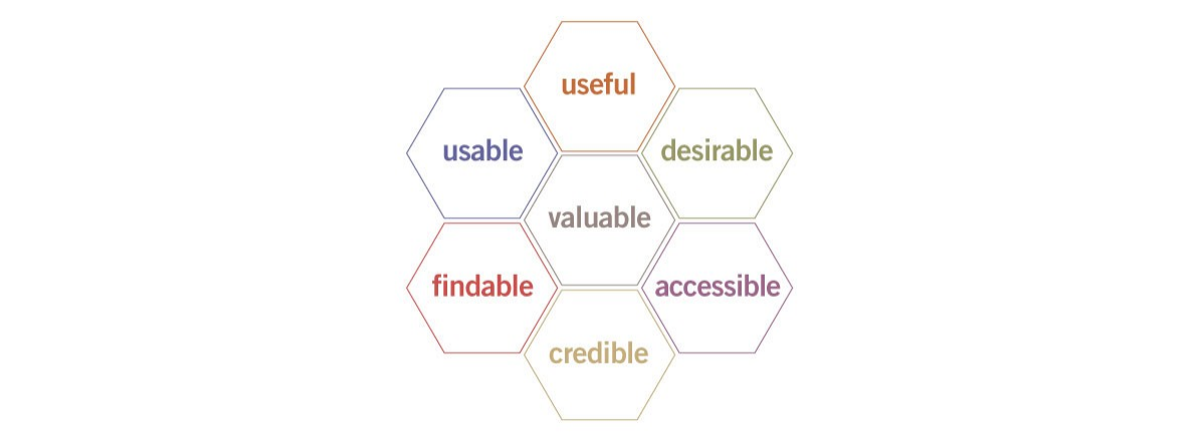
The honeycomb model of user experience.

Morville’s Honeycomb Model of User Experience Design [13] (Figure 2) was used to design the questionnaire and interview questions to properly assess the usability of the KT tools and identify ways to improve them. The honeycomb model breaks user experience into seven categories (usability, credibility, usefulness, desirability, findability, value, and accessibility) that can be used to categorize the various aspects of a user-friendly system and has been successfully used and validated by several past KT usability studies to design interviews and organize results [7,14,15].

### Sampling and Recruitment

Participants were eligible for enrollment into the study if they currently were a parent, guardian, or grandparent of a child under 18 years old, were 18 years or older themselves, and could read and speak English. The study was advertised via email to our current Pediatric Parent Advisory Group members and members of the Edmonton Early Childhood Coalition, as well as via Facebook, Twitter, and word of mouth throughout our local community. Ethics approval was received from our institutional ethics board, and all participants gave informed consent before any data collection.

### Study Components

#### Knowledge Questions and Structured Questionnaire

The knowledge test included six short answer questions to assess the effectiveness of the KT tool at accurately communicating health evidence (Multimedia Appendix 2). The participants were also asked to indicate the confidence in their responses using a 5-point Likert scale (very unsure, a little unsure, neither sure nor unsure, a little sure, and very sure) [8]. The questionnaire also asked a series of usability-related questions using an 11-point anchored Likert scale to obtain quantitative usability data regarding the individual KT tools.

#### Semistructured Interview

The third component of the study was a semistructured interview (Multimedia Appendix 1) consisting of two parts. The first part of the interview focused on the usability of the tool they were randomly assigned. Participants were then asked to read the other two tools. The second part of the interview asked the participants to compare the three KT tools and asked more general questions about KT tool preferences (eg, “What are the key parts of an ideal KT tool?” and “How much information is needed about the condition?”). The interview was field tested and adapted over three nonrecorded interviews using in-house parent volunteers.

#### Child Health Topic

Antibiotic use to treat AOM in children was selected as the health topic for the study because of the availability of a recent Cochrane review [16], the high prevalence and disease burden of AOM [17,18], high use of antibiotics to treat uncomplicated AOM despite best evidence [19,20], parental misinformation for treatment [21,22], and the identification of AOM as a priority for parental education from previous studies [22,23].

#### Knowledge Translation Tools Evaluated

A Cochrane PLS [16], Blogshot [24], and Systematic EvidEnce Disseminator (SEED)–updated Wikipedia page [25] on the use of antibiotics to treat AOM in children were recently developed or updated (Multimedia Appendix 1). These tools are publicly accessible over Web, the specifics of which are outlined below.

##### Plain Language Summary

Cochrane PLS were developed as a standalone summary of Cochrane systematic review findings aimed directly at health care consumers and written in plain language [26]. All Cochrane reviews must have a PLS freely available on Cochrane’s website and are required to follow a set of guidelines aimed to make them easy for the public to understand [27].

##### Blogshot

Cochrane Blogshots were originally developed by Cochrane UK as another method to effectively communicate recent Cochrane systematic reviews to consumers through social media [28]. Blogshots aim to present the key points in the Cochrane review relevant to consumers in a concise picture that can easily be shared on social media. Cochrane Child Health developed a blog, which houses the Blogshots and describes the results of the corresponding Cochrane review in an easy to read and less formal tone [24]. They are also shared on the Cochrane Child Health Facebook and Twitter accounts.

##### Systematic EvidEnce Disseminator–Updated Wikipedia Page

The Cochrane-Wikipedia partnership was formalized in 2014 with the aim to include relevant evidence and ensure the accuracy of all Wikipedia medical articles. Recently a novel software, SEED, has been developed to automatically generate summary of findings like tables compatible with Wikipedia from Cochrane Review Manager files [29]. These tables aim to present Cochrane review findings with a short description of the medical context in a way that is understandable and relevant to for health care consumers.

### Statistical Analysis

Knowledge test scores, KT tool reading times, and written questionnaire responses were treated as continuous data, and analyzed using a Kruskal-Wallis test because of failure to meet the normality and homogeneity of variance assumptions required for a three-way analysis of variance test. Knowledge test *confidence* scores were treated as ordinal data and analyzed via a Kruskal-Wallis test to identify a difference in confidence between groups. All analyses were performed using SPSS (IBM Corp) or Excel (Microsoft), considering a critical significance value *P*=.05.

Rank data from the semistructured interview were analyzed via Friedman test with a post hoc Wilcoxon signed-rank test to determine if any KT tool consistently ranked better in a given category. For post hoc testing among the three groups, a Bonferroni correction was applied resulting in a corrected significance value of *P*=.02.

### Thematic Analysis

Thematic analysis was used to synthesize and identify common parent preferences described in the semistructured interviews. Data management and analysis were facilitated using NVivo 12 Software (v.12, 2017 QSR International PTY Ltd.). Our process of thematic analysis followed the method outlined by Braun and Clarke [30]: familiarization with the data, initial coding, searching for themes among the initial codes, reviewing themes that may fit together as subthemes, and, then, defining and naming final major themes that best represented the data.

Data collection and analysis occurred iteratively, allowing for more precise and purposeful data collection. Data collection continued until saturation of major thematic categories was achieved. In our case, this was 16 interviews. Interviews were coded and categorized to facilitate development of themes. An inductive *bottom up* approach was taken, aiming to strongly link the developed codes and themes to the data themselves. The interviewer became immersed in the data through transcription of the recorded interviews. Initial coding for the first seven interviews was conducted using a *line-by-line* approach. Codes stayed close to the data by using participants’ own words as much as possible. Focused coding, grouping similar codes together, was then used to identify patterns in the data. These focused codes developed from the first seven interviews were then used directly for the remaining eight interview transcripts. To reduce interpretive bias, a second reviewer coded and categorized a random sample of 50% (7/15) of the interview transcripts. Any discrepancies between the reviewers were discussed and resolved via consensus. The focused codes were further refined via collaboration between the 2 reviewers into themes and subthemes that identified common factors contributing to parental preference and usability. All codes and transcripts were then re-examined to ensure consistency and accuracy of the interpretation.

### Recommendations

Recommendations for developing KT tools are proposed based on consensus of the authors using data gathered from questionnaire and parent interviews. These recommendations considered the core and subthemes of parent preferences identified through thematic analysis, trends in the questionnaire results, and the authors’ interpretation of the raw interview transcripts. These recommendations serve to create actionable items for researchers developing KT tools but represent a subjective interpretation of the data from the authors.

## Results

### Study Participants

The usability of three Cochrane KT tools on AOM was assessed by 16 parents. Participant demographics are presented in Table 1. Briefly, all participants were parents or grandparents, with 56% (9/15) of respondents being female. All participants had at least a high school diploma, with 88% (14/16) having a postsecondary degree equivalent or higher. In all, 25% (4/16) of participants self-reported that their child has had AOM in the past, and 19% (3/16) worked in the health care field. Finally, 63% (10/16) had heard of Cochrane before participation in the study, but many were unfamiliar with what research activities Cochrane Child Health and the Cochrane organization carry out.

**Table 1:** Demographic characteristics of participants in the study randomly assigned to each knowledge translation tool.

Characteristic		Knowledge translation tool evaluated				Total (N=16)
		PLS^a^ (n=5)	Blog^b^ (n=5)	Wiki^c^ (n=6)		
**Gender, n**						
	Female	3	3	3	9	
	Male	2	2	3	7	
**Parents’ age (years), n**						
	20-30	3	1	0	4	
	31-40	0	3	2	5	
	41-50	1	1	3	5	
	51+	1	0	1	2	
**Number of children, n**						
	1	3	2	2	7	
	2	2	2	2	6	
	3	0	1	1	2	
	4	0	0	1	1	
**Highest level of education, n**						
	High school diploma	0	0	0	0	
	Some postsecondary	1	0	1	2	
	Postsecondary degree	1	2	1	4	
	Master’s	2	2	4	8	
	PhD	1	1	0	2	
**Works in health care, n**						
	Yes	0	1	2	3	
**Heard of Cochrane reviews, n**						
	Yes	3	2	5	10	
**Child has had AOM^d^, n**						
	Yes	3	0	1	4	

^a^PLS: plain language summary.

^b^Blog: Blogshot.

^c^Wiki: Wikipedia page.

^d^AOM: acute otitis media.

### Effectiveness of Tested Knowledge Translation Tools

We assessed efficiency through timing parents reading the KT tool and effectiveness through a short answer knowledge retention test in addition to parents self-reported confidence in their answers.

**Figure 3:**
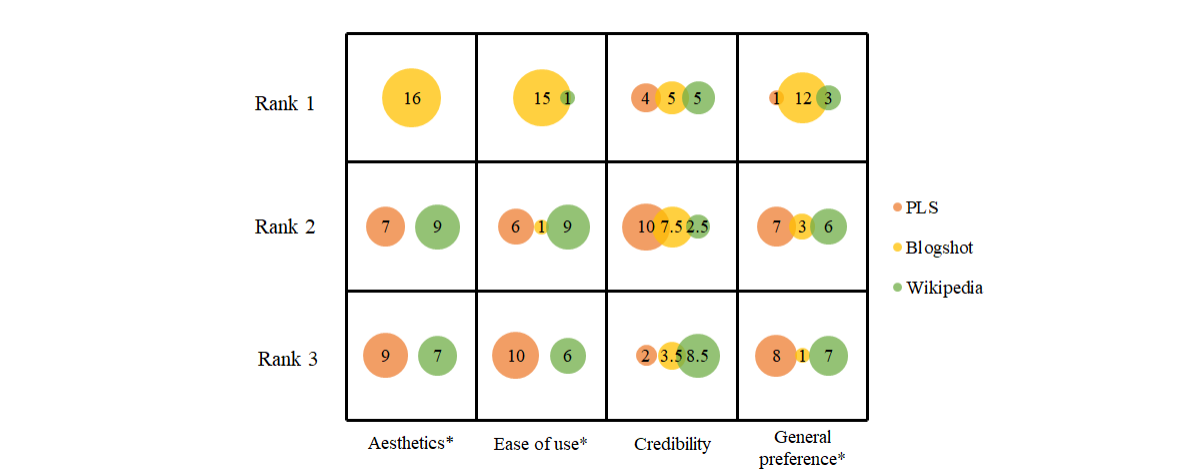
Plain language summary, Blogshot, and Wikipedia ranked in order of most (Rank 1) to least (Rank 3) in categories of aesthetics, ease of use, credibility, and general preference. Dot area indicates number of participants placing each tool at a given rank (N=16). Asterisk indicates statistically significant difference in rank order between groups determined via a Friedman test (*P*<.05). PLS: plain language summary.

Parents on average spent less time (mean, range) reading the PLS (163 seconds, 129-200 seconds) and Blogshot (168 seconds, 97-257 seconds) than the updated Wikipedia page (275 seconds, 91-519 seconds); however, there was no significant difference among the three groups, χ^2^_2_=3.2, *P*=.20 (Table 2, Figure 3). Although parents spent the most time reading the Wikipedia article, the Wikipedia page also had the shortest and longest reading times, 92 seconds and 519 seconds, respectively. There was no difference in efficiency among the three KT tools.

**Table 2:** Summary of quantitative data from knowledge test and usability questionnaire (Q1-Q9 refer to usability questionnaire included in Multimedia Appendix 2. Likert scales have been flipped so a higher number is always better).

Variables assessed		Knowledge translation tool			*P* value (Kruskal-Wallis)
		PLS^a^	Blogshot	Wikipedia	
Sample size (N=16), n		5	5	6	N/A^b^
Time reading tool (seconds), mean (95% CI)		163 (127-200)	168 (91-244)	275 (114-435)	.20
Knowledge test score, mean (95% CI)		4.2 (2.4-6.0)	4.6 (3.2-6.0)	3.7 (3.1-4.2)	.31
**Survey responses, mean (95% CI)**					
	Q1 Key information	6.2 (3.0-9.4)	8.2 (6.2-10.0)	7.8 (6.0-9.6)	.44
	Q2 Easy to remember	3.6 (0.4-6.8)	7.2 (4.2-10.0)	3.8 (0.84-6.8)	.08
	Q3 Increased knowledge	8.2 (6.2-10.0)	8.6 (6.9-10.0)	6.5 (2.7-10.0)	.54
	Q4 Aesthetically pleasing	4.8 (1.7-7.9)	6.8 (3.4-10.0)	2.5 (0.3-4.7)	.07
	Q5 Mentally demanding	2.6 (1.2-4.0)	5.4 (2.2-8.6)	3.2 (1.8-4.6)	.11
	Q6 Understandable	5.6 (3.2-8.0)	7.6 (5.3-9.9)	7.7 (5.0-10.0)	.16
	Q7 Frustrating to read	4.4 (1.2-7.6)	7.8 (5.0-10.0)	4.5 (0.9-8.1)	.11
	Q8 Helps with decision making	6.6 (1.7-10.0)	7.6 (4.2-10.0)	5.7 (4.1-7.2)	.32
	Q9 Would recommend to others	4.8 (0.38-9.2)	7 (3.7-10.0)	3.7 (0.8-6.5)	.20

^a^PLS: plain language summary.

^b^Not applicable.

Although there was no difference among groups in knowledge test scores (χ^2^_2_=2.4, *P*=.31), parents on average answered the most questions correctly after reading the Blogshot (4.6/6), followed by the PLS (4.2/6) and Wikipedia page (3.7/6). Parents who were assigned the Blogshot reported feeling slightly more confident in their responses; however, this was not significantly different across groups.

### Identifying Knowledge Translation Tool Preference

Parents’ ranking results of the three tools for aesthetics, ease of use, credibility, and general preference are presented in Figure 3. There was a significant difference in the ranking of aesthetics (χ^2^_2_=24.1, *P*<.001), ease of use (χ^2^_2_=21.9, *P*≤.001), and general preference (χ^2^_2_=11.6, *P*=.003) among the three tools. Post hoc analysis showed that parents preferred the Blogshot over the PLS or Wikipedia page in the categories of aesthetics (*P*<.001 and *P*<.001, respectively), ease of use (*P*<.001 and *P*=.001, respectively), and general preference (*P*=.004 and *P*=.011, respectively). There was no significant difference in the ranking between the PLS and Wikipedia page in any category. There was no statistically significant difference in the ranking of the credibility of the three tools (χ^2^_2_=1.4, *P*=.50). Overall, parents found the Cochrane Blogshot to be the most preferred, the most aesthetic, and the easiest tool to use.

### Identifying Factors Leading to Parental Preferences

Survey responses did not show any significant difference in parent preferences among the three KT tools (Table 2). However, the Blogshot was ranked highest in all categories except understandability, where participants narrowly preferred the updated Wikipedia page (7.6 vs 7.7, respectively).

However, four themes were identified through thematic analysis relating to parental preferences (Table 3). A detailed explanation on how we reached these themes is included in the Methods section. In brief, parents want a tool that is (1) simple, (2) trustworthy, and (3) quick to access and use, and which (4) informs how to manage the condition. A detailed description of each theme and supportive illustrative quotes are documented in Multimedia Appendix 2.

**Table 3:** Themes and subthemes of parental knowledge translation tool preferences.

Theme	Subthemes
Simple	Understandable language Nonmedical graphics Simple and familiar aesthetic Not interested in study characteristics
Trustworthy	Evidence based Recognized source Cites sources Peer advice
Quick to access and use	Easy-to-find electronic tools Efficient organization Concise Usable in stressful scenarios
Informs how to manage the condition	Describes what to expect Explains when to seek care Describes how to manage the child at home

### Recommendations

These recommendations (Table 4) take into account both the quantitative and qualitative observations from the gathered data and should be considered when developing child health–related KT tools for parents or caregivers. A detailed explanation for how we arrived at these recommendations can be found in the Methods section.

**Table 4:** Checklist of recommendations for developing child health knowledge translation tools for parents or caregivers.

Elements	Recommendation
Content	Introduction should contain symptoms, so parents can determine if the article is relevant. Include information on how to manage child at home, when to seek care, and what to expect. Only include statistics from the research if they are meaningful to a caregiver not familiar with the condition.
Format	Use headings and bullet points to offer a familiar and efficient organization. Replace words with graphics wherever possible. Be concise, but link to more information. More details confuse parents and reduce readership.
Language	Use repetition selectively and sparingly. Avoid abbreviations, jargon, and polysyllable words to make writing easy to understand. Write as if you are speaking to a stressed parent.
Branding	Associate with a recognized brand to increase credibility and findability.

## Discussion

### Principal Findings

We found no significant differences among the PLS, Blogshot, and Wikipedia pages in terms of reading time and knowledge retention. This finding is consistent with a pervasive difficulty in the KT field of identifying quantitative differences in the effectiveness of different KT tools [2]. An identified flaw contributing to this problem is the lack of adequate power in many studies to identify statistically significant differences. Often, as in our case, the desire to include time-intensive interviews and qualitative analysis makes reaching such statistical power unachievable. Future studies interested in the quantitative differences between tools may consider eliminating or limiting qualitative analysis to sufficiently reach those goals.

Using the honeycomb model of user experience to describe usability, we conducted a comparative usability analysis of three Web-based KT tools. We determined that parents prefer Blogshots as a KT tool compared with the PLS and updated Wikipedia pages, and ultimately found Blogshots to be the most usable tool. Usability remains difficult to define universally in the KT field. Although many previous studies have similarly defined usability through a model such as the honeycomb model of user experience [7,14,15], others have defined it to meet specific objectives of their research [5,31]. Without an agreed upon model of usability within the KT field, the definition ultimately depends on how it is measured in each individual study [32]. Unfortunately, measurement techniques vary significantly between studies as well, often leading to significant bias and results of questionable utility to the larger field [2,32]. As KT usability research continues to develop, it will be vital to move toward a standard definition to allow research with broad generalizable implications to be realized.

Through thematic analysis of interview transcripts, we revealed four emergent themes: parents want KT tools that are (1) simple, (2) easy to access and use, and (3) trustworthy, and which (4) inform how to manage the condition. Although these themes seem simple to understand and implement, many are lacking from currently used pediatric KT tools. Previous KT studies using thematic analysis focused on a single tool, using thematic analysis as a way of recognizing usability hurdles specific to that tool [7,8,14,33]. To our knowledge, no previous study has identified themes leading to KT tool preference and usability that are applicable to a wider range of KT tools. We hope that by using a comparative usability methodology, these themes will be more broadly generalizable to the larger pediatric KT field. Furthermore, using all the data collected and interviews conducted, we developed a set of recommendations for developing KT tools directed at parents. These findings were consistent with previously identified weaknesses in the usability of the Cochrane Library as a whole for health professionals [15].

Through a comparative usability analysis, we present a novel method of identifying factors contributing to usability that are more generalizable than previous KT tool–specific studies. Comparative usability analysis is different from traditional comparative studies where participants are assigned to 1 of typically 2 tools and quantitatively compared. In comparative usability analysis, participants are shown all KT tools and asked to identify what factors, shared or unique, about elements they preferred or disliked. By showing participants many ways of presenting the same information, we theorize that it primes participants to think about new ways of presenting the same material, and can lead to more creative and insightful feedback. While achieving useful insights about each tool, this method also contributes to our understanding of the universal themes leading to parental preferences and ultimately determining the degree of usability across all KT tools. We hypothesize that our recommendations are more methodologically rigorous than conducting a similar analysis where participants are shown only a single tool, yet further studies are needed to develop the comparative usability analysis method in the field of KT.

Another interesting result in our study was that the participants ranked the three KT tools as being similarly credible. We theorized that Wikipedia would be ranked lowest in credibility because of our perceived common understanding that it is editable by anyone. Credibility, however, was very difficult for participants to assess and rank among the three tools. Interestingly, it was difficult to assess not because people’s perception of the tools’ credibility varied significantly, but because most viewed all three tools as being very credible. Participants often cited familiarity with the source, such as Wikipedia, or traits that make it look official, such as citations, complex terms, and familiar sources contributing to their perception of credibility. Similarly, previous research into credibility identified that consumers chiefly make credibility judgments based on factors visually prominent on the page such as aesthetics and brand recognition [34,35]. With the large availability of poor-quality information over Web, this contributes to the potential of parents accessing misinformation unintentionally. It also further emphasizes the importance of disseminating quality KT tools to consumers to compete with well-designed, but factually incorrect, Web-based health information.

### Limitations

Despite having good representation across age groups, number of children cared for, and both mothers and fathers, our sample size and selection was the largest limiting factor in our study. Owing to the qualitative components of the study, we were limited to a relatively small sample size (N=16), which restricts the utility of the quantitative results. It is possible that with a larger sample size, we may have identified statistically significant differences among the effectiveness and efficiency of the three KT tools. Future quantitative studies with larger sample sizes are still needed to address the difference of effectiveness among the three tools. However, our sample size allowed for a more robust and bottom-up approach to the qualitative analysis reducing bias in our interpretation. We found that the interviews were very similar between individuals with the four identified themes being touched on in nearly every interview.

Our sample also consisted of mostly highly educated individuals, with 88% (14/16) participants having a postsecondary degree or higher. Although it is possible that parents or guardians with a lower level of education may prefer information presented differently, some of these concerns were reduced by the participants indicating that they desired simplicity and less technical information.

Finally, as the sample consisting of only parents or guardians, the results should be interpreted with caution for developing adult health care KT tools. Although many of the themes and preferences may be similar, the difference between adult and pediatric medicine, as well as the different dynamics between being a caregiver and a patient, may significantly change what an individual would want from a KT tool. Furthermore, we only conducted research on a single acute condition, and the findings may not be applicable to more urgent or chronic pediatric conditions.

### Conclusions

The comparison of three Web-based KT tools on the same pediatric health topic allowed us to identify four underlying themes parents want from a KT tool: simple, easy to access and use, trustworthy, and to be able inform how to manage the condition. The development and exploration of subthemes provide meaningful insight into how to achieve these simple but hard to reach objectives when translating health evidence for general consumption. We identified that Blogshots are the preferred (from the three tools tested) KT tool for parents, and using research consensus, these findings have been translated into a checklist to consider when developing a KT tool aimed at parents and familial caregivers. Our research provides meaningful insight for developing and improving future pediatric KT tools. Further usability testing of different KT tools should be conducted involving broader populations, other conditions (eg, acute vs chronic), and varying decision-making needs to generate guidelines to improve KT tools for parents, ultimately improving child health outcomes.

## Appendix

Multimedia Appendix 1 Interview package containing participant instructions, knowledge questions, usability questionnaire, and semi-structured interview prompts.

Multimedia Appendix 2 Subtheme description and illustrative quotes for parental knowledge translation tool preference.

## Abbreviations

AOM: acute otitis media
KT: knowledge translation
PLS: plain language summary
SEED: Systematic EvidEnce Disseminator
