# Supplementary material for: Comparative Usability Analysis and Parental Preferences of Three Web-Based Knowledge Translation Tools: Multimethod Study
Source: J Med Internet Res. 2020 Mar 13;22(3):e14562. doi: 10.2196/14562 (PMC7101501; doi:10.2196/14562)
Supplement: Multimedia Appendix 1 [file jmir_v22i3e14562_app1.docx]

## Multimedia Appendix 1

**Sample Participant Instructions:**

Scenario:

You are a parent of a 4-year-old child who you suspect has an ear infection. You want to know more about ear infections before seeking care and after a google search find the **Wikipedia** page titled “Otitis Media”.

It is your job to use this page as you think a parent looking to help their child would in this scenario. Specifically, look for information to answer the following questions that may be on a parent’s mind:

**What is some general information about ear infections?**

**If my child has an ear infection, how will they be treated?**

**What are the benefits and risks to this treatment?**

**You do not need to read the entire Wikipedia page. You may skim or skip over what you think is not relevant to the scenario.**

When the interviewer says to begin, you may look at the **Wikipedia** page on the iPad provided. You have as long as you want to look at the web page. When you feel you have all the information you need from the web page let the interviewer know and the next part of the interview will begin.

**Please only read the Wikipedia page.**

**Do not flip this page until instructed**

**Instructions:**

**This section aims to assess how effective the knowledge translation tool you just read is at communicating health care information. Please answer questions to the best of your ability, and indicate your level of confidence in your response.**

**What is Acute Otitis Media?**

________________________________________________________________________________________________________________________________________________________________________________________________________________________________________________________________________________________________

Very unsure A little unsure neither sure/ A little sure Very sure

nor unsure

**What are the signs and symptoms of Acute Otitis Media?**

________________________________________________________________________________________________________________________________________________________________________________________________________________________________________________________________________________________________

Very unsure A little unsure neither sure/ A little sure Very sure

nor unsure

**What are the benefits to using antibiotics to treat Acute Otitis Media?**

________________________________________________________________________________________________________________________________________________________________________________________________________________________________________________________________________________________________

Very unsure A little unsure neither sure/ A little sure Very sure

nor unsure

**What are the harms to using antibiotics to treat Acute Otitis Media?**

________________________________________________________________________________________________________________________________________________________________________________________________________________________________________________________________________________________________

Very unsure A little unsure neither sure/ A little sure Very sure

nor unsure

**Do all children with Acute Otitis Media need antibiotics?**

________________________________________________________________________________________________________________________________________________________________________________________________________________________________________________________________________________________________

Very unsure A little unsure neither sure/ A little sure Very sure

nor unsure

**What is the preferred method for treating mild-moderate Acute Otitis Media in children?**

________________________________________________________________________________________________________________________________________________________________________________________________________________________________________________________________________________________________

Very unsure A little unsure neither sure/ A little sure Very sure

nor unsure

**This section asks some questions about how easy the knowledge translation (KT) tool was to use. Please circle your response and add any details/explanation if prompted. Please be honest in your answers, the interviewer did not design these tools and is looking for honest, constructive feedback. If you have additional comments, you may write it down or tell the interviewer.**

1. Does the KT tool provide **key information** about ear infections that is relevant to parents? *Please circle your response.*

Yes, all key information is provided

No, important key

information is missing.


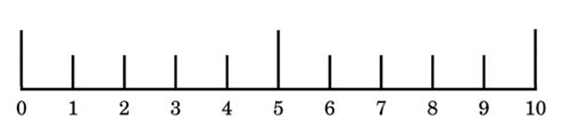


Comments: ________________________________________________________________________________________________________________________________________________________________________________________________________________________

1. Do you think this method of information presentation makes it **easy to remember** the information? *Please circle your response.*

Yes, the information is easy to remember

No, the information is hard to remember


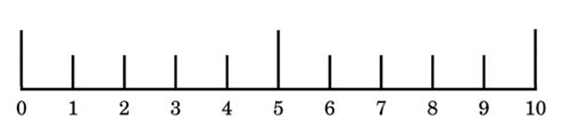


Comments: ________________________________________________________________________________________________________________________________________________________________________________________________________________________

1. Did you feel the KT tool **increased your knowledge** about the treatment of ear aches in children? *Please circle your response.*

Yes, I feel like I know significantly more about the topic

No, I do not feel like I know significantly more about the topic


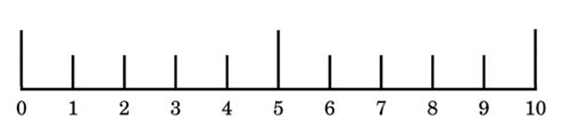


Comments: Comments: ________________________________________________________________________________________________________________________________________________________________________________________________________________________

1. Is the KT tool **aesthetically pleasing (i.e., images, colours, headings, etc.)**? *Please circle your response.*

No, the KT tool is

distasteful/unattractive

Yes, the KT tool is tasteful/ attractive


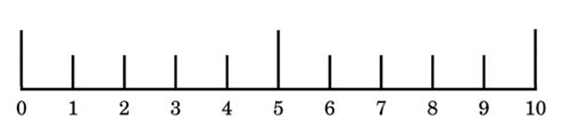


Comments: Comments: ________________________________________________________________________________________________________________________________________________________________________________________________________________________

1. Was reading the KT tool **mentally demanding?** *Please circle your response.*

No, the KT tool took little mental effort to read

Yes, the KT tool took lots of mental effort to read


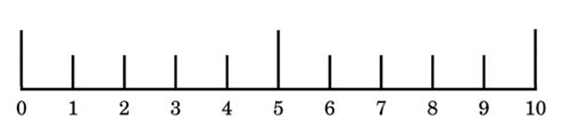


Comments: Comments: ________________________________________________________________________________________________________________________________________________________________________________________________________________________

1. Is the KT tool **understandable?** *Please circle your response.*

No, it was confusing/hard to understand

Yes, it was clear and easy to understand readread/undesrs


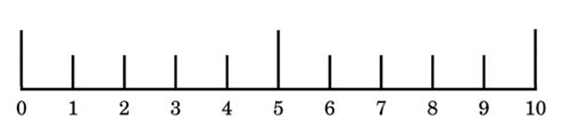


Comments: Comments: ________________________________________________________________________________________________________________________________________________________________________________________________________________________

Was reading the KT tool ever **frustrating?** *Please circle your response.*

Yes, I found the format frustrating

No, I found the format pleasant


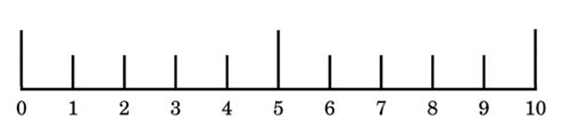


Comments: ________________________________________________________________________________________________________________________________________________________________________________________________________________________

1. Would you **use** this KT tool **to help you make decisions about your child’s health**? *Please circle your response.*

Yes, I would use this tool

No, I would never use this tool


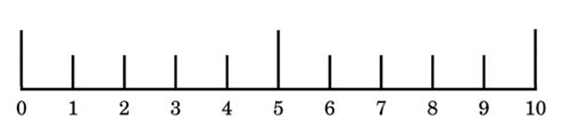


Comments: ________________________________________________________________________________________________________________________________________________________________________________________________________________________

1. Would you **recommend** this KT tool to parents/families you know to help them **make decisions** about their **child’s health**? *Please circle your response.*


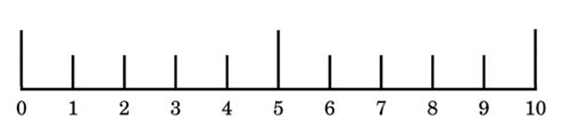


No, I would never recommend this tool

Yes, I would always recommend this tool

Comments: Comments: ________________________________________________________________________________________________________________________________________________________________________________________________________________________

**Thank you for your feedback!**

**Tool Focused Semi-Structured Interview**

**Only do one of the KT tool specific question lines based on which tool the participant is randomly assigned to.**

**Blogshot questions:**

- What is one thing you liked about the blogshot?
- What is one thing you would change about the blogshot?
- Did you find this KT tool organized effectively?
- Was information you cared about easy to find within this KT tool?
- Do you find the information within this tool credible? Why or why not.
- Do you know where to find blogshots on the internet?
- Do you see any barriers someone may have to using this tool? Text size, font, readability, language?
- What would you title the blogshot so other parents would read it upon seeing it in their social media feed?

**Cochrane PLS Questions:**

- What is one thing you liked about the Cochrane PLS?
- What is one thing you would change about the Cochrane PLS
- Did you find this KT tool organized effectively?
- Was information you cared about easy to find within this KT tool?
- Do you find the information within this tool credible? Why or why not.
  - - Do you know what the Cochrane Collaboration is?
- Do you know where to find Cochrane PLS on the internet?
- Do you see any barriers someone may have to using this tool? Text size, font, readability, language?

**Wikipedia questions:**

- What is one thing you liked about the Wikipedia page?
- What is one thing you would change about the Wikipedia page?
- Did you find this KT tool organized effectively?
- Was information you cared about easy to find within this KT tool?
- Do you find the information within this tool credible? Why or why not.
- Do you know where to find Wikipedia pages on the internet?
- Do you see any barriers someone may have to using this tool? Text size, font, readability, language?
- *Interviewer will draw attention to the SEED table in the Wikipedia article*
  - Did you find the SEED table in the Wikipedia article helpful?
  - Did you find the table understandable?
  - What did you like about the table?
  - What would you change about the table?
  - Do you find the table aesthetically pleasing?
  - Do you find the summary helpful?
  - Do you understand the “Finding in numbers” column?
    - Do you know what RR means?
    - Relative Risk?
  - Do you understand the title of the table?
  - Do you understand where this table comes from?
  - What, if any, information should be provided with the table to make it easier to understand?

*************Participants shown other two tools*************

**General/Comparative Semi-Structured Interview**

Which, of the KT tools did you find most useful?

Could you see yourself using any of these three KT tools to make health care decisions about your children?

- What factors are most important to making the KT tools useful to parents?

What do you think would be the most useful way to present information?

- What format do you think would best meet the needs of parents?
- Do you think we need to show all data?
- Are tables helpful?
- Are pictures helpful?
- Are statistics helpful?

What do you think are key parts of a KT summary product?

- What information do you think is needed about AOM as a minimum?
- Can there be too much information? When do you think that would be?

Where do you think would be the best place for parents to find information about child health topics?

- Do you think parents would go to Wikipedia pages for child health information?
- Do you think parents would go to social media platforms (Facebook, twitter) for health information?
- Do you think parents would go to Cochrane Reviews for health information?
  - - Do you know what the Cochrane Collaboration is?
- Do you commonly have access to the internet when posed with a child health question?
  - Do you often have time to look up your questions?

Do you understand what is meant by “Quality of evidence”?

- What does high quality evidence mean?
- What does very low quality evidence mean?

Please rank the three KT tools in order of most to least aesthetically pleasing.

- Is there any specific reason for this ranking?

Please rank the three KT tools in order of ease of use

- Is there any specific reason for this ranking?

Please rank the three KT tools in order of credibility

- Is there any specific reason for this ranking?

Please rank the three KT tools in order of general preference

- Is there any specific reason for this ranking?

Do you have any other comments or thoughts about these KT summary products? Or suggestions for how to improve them?
