# Supplementary material for: Comparative Usability Analysis and Parental Preferences of Three Web-Based Knowledge Translation Tools: Multimethod Study
Source: J Med Internet Res. 2020 Mar 13;22(3):e14562. doi: 10.2196/14562 (PMC7101501; doi:10.2196/14562)
Supplement: Multimedia Appendix 2 [file jmir_v22i3e14562_app2.docx]

## Multimedia Appendix 2

| **Themes and subthemes of parental KT tool preferences** | | | |  |
| --- | --- | --- | --- | --- |
| **Theme** | | **Subthemes** | **Description** | **Illustrative Quote** |
| **Simple** | - Understandable language | | - Parents want to be able to read the tool and understand it with minimal effort. To remain simple, KT tools should avoid jargon, acronyms, polysyllable words, and in-text citations. | “When compared with placebo for acute otitis media in children’. Well Jesus if I am a parent I am not wanting to write a dissertation on this…. But meanwhile all it does is just ah parade the authors, the authors credential and impede the ah clients learning” |
|  | - Non-medical graphics | | - Parents identified that pictures assisted in breaking up the text, simplifying the aesthetics and readability. Further, images can significantly aid understanding if used effectively. Parents also highlighted that photos of medical conditions not visible to the naked eye are largely unhelpful and are a source of anxiety for some. | “it breaks up the information and makes it less boring to read.so.. if its…if it just text.. um.. your eyes are naturally going to go to the picture. And then if you have the information in the picture, um… I pick it up better”  “they (photos of tympanic membrane) are freaky. I don’t understand what they are. They all look like strange pupils or slugs or…They are awful images. And I am sure they are interesting to someone, but not to me” |
|  | - Simple and familiar aesthetic | | - Parents find tools that they are already familiar with to be easier to use and to find information within. | “for better or worse a lot of us will probably go to Wikipedia because we are so used to it. Like we know how it is laid out huh, you can kind of get the information you need” |
|  | - Not interested in study characteristics | | - While parents like reading simplified results, demographic information and statistical analysis add confusion and decrease the clarity of communication. | “I really like the 60% get better within 24 hours because that shows you, oh the majority do get better and they actually don’t have to go to the doctor”  “like for example I don’t need to know the 95% confidence interval. Um I only learned what a 95% confidence interval was in my PhD. So I really don’t think most parents would be like “oh great, the confidence interval is 1.24 to 2.36, I feel so good about that” |
| **Trustworthy** | - Evidence based | | - The perception that a tool is evidence based increases the credibility of a tool for parents. This perception comes from either the term “evidence based” in the tool or statistics included in the tool. | “Obviously there is lots of information out there on the internet right? I could look at if my kid has an infection. But, knowing this is actually based on research was quite important for me to, to continue reading. And to trust.” |
|  | - Recognized source | | - The source of the KT tool was a significant contributor to the perceived credibility of a tool. Parents tended to rank sources they recognized, such as Wikipedia, higher than sources like Cochrane that they did not recognize. | “I’d say, everyone knows Wikipedia, so you just assume that it is credible I guess.” |
|  | - Cites sources | | - The act of including cited sources at the bottom of the tool improved the perceived credibility. | “after all, there are 59 footnotes, must be credible” |
|  | - Peer advice | | - Parents noted anecdotal advice from parents/caregivers who have experienced the illness before as an important and credible source when making decisions about their children’s healthcare. | “if I wanted to know from a parent who has gone through something like this I would probably call them up…I am just kind of old school” |
| **Quick to access and use** | - Easy to find electronic tools | | - Parents want tools that are easy to find when they need them. Typically, parents will google the chief complaint of the child and use one of the first results. | “I would google “ear infections in kids” is probably what I would google. And whatever shows up first is probably where I would go to” |
|  | - Efficient organization | | - Efficient organization was indicated as one of the most effective ways to make a tool quick to use. The key organization techniques desired by parents are starting with symptoms so they can determine if the article is applicable right away. Furthermore, parents value bullet points to make skimming easier and condense information. Headers also aid in skimming and add to an efficient organization. | “I would start with, like, symptoms, and then background.[..] I always search for the symptoms in bullet points and then I see if, if that’s what my son has symptoms of that, then I read the article. If not, I am not going to read it.”  “Maybe make some bullet points instead of big paragraphs ah, bullet point are easier to, to skim through and maybe bold a few key words”  “The headings are good. Because you look quickly and it is like “ok what am I looking at” there’s the title there’s the background, and then, this is what the evidence says. […] So it’s very organized that way.” |
|  | - Concise | | - One of the key parts to making a tool quick to use is by condensing information. While the organization aids in condensing information, parents generally felt that some background information and other portions of the tool could be removed without sacrificing quality. Some parents mentioned cutting down on repetition between summaries and body text to condense the tool. | “I would cut to the chase. Um…I would take out review questions, background, study characteristics”  “just in general I would get rid of half the content”  “If I find so much repetition on any information, … I, I lose my patience and I think oh this is a bad page” |
|  | - Usable in stressful scenarios | | - Unique to parents, participants mentioned when accessing KT tools on the internet they are often significantly stressed by the illness of their child. They felt a parent focused tool should keep the stressed state of parents in mind. | “I would throw it [the KT tool] entirely away and start over by writing something that seems to be [addressed] to an audience of, anxious parents”  “The other thing too is I think you have to consider like…when my kids crying and I am, I am in distress because my child is in distress. I don’t want to read….like, I am literally just like, I’m just like, I am almost panicked.” |
| **Informs how to manage the condition** | - Describes what to expect | | - Once parents know they need to go to the Emergency Room or family physician, they reported wanting to know what to expect from that visit. Parents indicated that having reasonable expectations before seeing the doctor makes it easier to accept a treatment they may not have expected such as watchful waiting instead of an antibiotic prescription. - Parents also wanted to know what kind of prognosis to expect. While parents liked knowing red flags to look out for, they indicated that rare complications create stress and are unnecessary to fulfill their needs as caregivers. | I think parents go to their family physician with the expectation that they will be given antibiotics, and that’s actually more the exception versus the rule. And so if parents have read this they may actually go get their child checked out for maybe reassurance sake but when they walk away with the physician having said try a warm compress with Tylenol and Advil alternating the parents aren’t disappointed”  “ when you are talking about very rare outcomes that are pretty extreme like hearing loss…or…you know ruptured ears those sorts of things I mean of course it’s good to know that…it’s possible that some of those things can happen... but... . I think you don’t want to cause people to be extremely afraid or…think that something is gonna... you know…something terrible is going to happen... um…so that might be kind of one line for…we know for most kids this is going to resolve after a couple of days, if not go to the doctor” |
|  | - Explains when to seek care | | - The first things parents wanted to know is if and when they should take their child to see a physician. Parents indicated commonly searching for information online to solely answer these questions. | “I want to know A: when do I need to see my family physician.. ahh what are the symptoms that present that prompt that type of visit. B: ah..When do symptoms escalate to a point where I need to make a trip to the emergency department?” |
|  | - Describes how to manage the child at home | | - Lastly, parents want to know what they can do at home to manage the condition and make their child feel better. | “there is nothing in here about home remedies in terms of what you can do in terms of making the child more comfortable. Um, They are basically saying that antibiotics don’t work. k, so what does? So their are lots of pieces missing in this” |
